# Secondary Metabolites and Eco-Friendly Techniques for Agricultural Weed/Pest Management

**DOI:** 10.3390/plants10071418

**Published:** 2021-07-12

**Authors:** Fabrizio Araniti, Marco Landi, Vito Armando Laudicina, Maria Rosa Abenavoli

**Affiliations:** 1 Dipartimento di Scienze Agrarie e Ambientali—Produzione, Territorio, Agroenergia, Università Statale di Milano, Via Celoria n°2, 20133 Milano, Italy; 2 Department of Agriculture, Food and Environment, University of Pisa, Via del Borghetto 80, 56124 Pisa, Italy; marco.landi@unipi.it; 3 Dipartimento di Scienze Agrarie, Alimentari e Forestali, Università degli Studi di Palermo, Viale delle Scienze, 90128 Palermo, Italy; laudicina@unipa.it; 4 Dipartimento Agraria, Università Mediterranea di Reggio Calabria, Località Feo di Vito SNC, 89124 Reggio Calabria, Italy; mrabenavoli@unirc.it

In agro-ecosystems, pests (insects, weeds, and other plant’s parasites) compete with crops for edaphic resources, negatively affecting quality and crop yields [1]. Nowadays, synthetic pesticides, easy to apply and accessible to farmers, are the most common and effective methods for pest management [2]. Nevertheless, the negative impact of these chemicals on the environment, human health, and the development of herbicides/pesticides-resistance are shifting the attention to alternative pest control technologies based on natural compounds [3,4,5,6]. Therefore, new eco-friendly agronomic techniques and the use of natural or natural-like molecules might represent a valid alternative strategy for pest control in the framework of sustainable agriculture [7,8,9].

The Special Issue “Secondary metabolites and eco-friendly techniques for agricultural weed/pest management” is timely and could offer interesting contributions to readers on the most recent aspects related to this pivotal topic. It includes 12 research papers (11 original articles and a scientific review) in which different aspects of pest management, from basic research to potential practical approaches, have been investigated through the latest and innovative technologies.

Three of the twelve published manuscripts are focused on the potential use of the essential oils (EOs) formulations for weed management [10,11,12].

Jouini et al. [10] deeply explored the phytotoxic effects of *Thymbra capitata* (L.) Cav., *Mentha × piperita* L. and *Santolina chamaecyparissus* L. EOs on several noxious weeds. Moreover, the authors investigated the effects of EOs distribution on soil microbial biomass carbon and nitrogen balance, microbial respiration, and microbial groups speciation. They concluded that *T. capitata* EOs was the most effective against all the selected weeds, whereas *P. oleracea* resulted the most resistant weed. Moreover, except for the EOs extracted from *T. capitata*, which did not allow soil microorganisms to recover their initial functionality, the other EOs initially inhibited soil microorganism development. However, after a brief period, the soil microbiota recovered its initial function and biomass. Thus, the authors concluded that EOs could be an excellent alternative to synthetic herbicides for weed management, but their optimal application doses must be carefully identified to control weeds without affecting soil microorganisms.

Similarly, Abd-ElGawad et al. [12] bioassayed in vitro the EOs extracted from *Argemone ochroleuca* Sweet, mainly constituted by oxygenated terpenoids, on noxious weed *Peganum harmala.* Their results confirmed the hypothesis that oxygenated monoterpenes were more phytotoxic than non-oxygenated ones [13]. Finally, Synowiec and Krajewska [11] demonstrated that maltodextrin microencapsulated peppermint oil mixed with silty clay loam soil substrate strongly inhibited the growth of wild mustard, suggesting that the microencapsulation could be an excellent strategy to formulate new bio-herbicides.

Among the published manuscripts, two focused on the mode of action of two natural compounds, nerolidol and norharmane, belonging to the terpenoid and indole alkaloids classes, respectively [14,15].

The sesquiterpenoid nerolidol was assayed in vitro on the model species *Arabidopsis thaliana*, and its mode of action was studied in depth through a metabolomic approach. The results highlighted that metabolomic-scale changes induced by nerolidol (in sugar, amino acid, carboxylic acid profiles and hormonal profile) supported the multi-target action of this molecule, which is a positive feature for a bio-herbicide [14]. Similarly, the experiments on norharmane investigated the specificity of this molecule on several crops and weeds (monocots and dicots). Moreover, the authors deeply evaluated its phytotoxicity on Arabidopsis adult plants using two different application modes (leaf spraying and watering). They finally demonstrated that norharmane differentially affected weed germination and growth (some species were more sensitive during germination and less during the growth and vice-versa). In contrast, on Arabidopsis, watering was the most effective way for norharmane application inducing water stress and compromising the photosynthetic machinery [15]

One of the main strategies for discovering new molecules with phytotoxic potential is classical extraction techniques such as maceration, distillation, and decoction, followed by a bio-guided fractionation and chemical characterization of the putative phytotoxic compounds. Nomura et al. [16] screened the 50 Chinese medicinal plants and their different organs, discovering that the fruits of *Illicium verum* Hook. f. (star anise) were the most phytotoxic and identified the shikimic acid (about 7% of star anise dry weight) as the main phytotoxic compound.

Similarly, Kapoor et al. [17] demonstrated that the aqueous extract of *Artemisia absinthium* and *Psidium guajava* significantly affected the noxious weed *Parthenium hysterophorus* inhibiting its germination and root growth, inducing lipid peroxidation and affecting seedling ROS scavenging activity.

One of the main problems in studying plant extracts as potential bio-herbicide is their impact on the environment because of the wide use of organic solvents such as methanol, chloroform, hexane etc., which are not healthy and environmentally safe. Therefore, in the last years, it has mainly been explored the potential use of green techniques to achieve biologically active extracts.

In this contest, Jiang et al. [18] used the supercritical CO_2_ extraction technique to extract the volatile fraction of *Baeckea frutescens*. They bioassayed the fumigant ability of this extract on *Colletotrichum gloeosporioides* and *Pseudopestalotiopsis*, two of the most important *Camellia sinensis* L. pathogenic fungi. Finally, they identified β-caryophyllene, α-caryophyllene, δ-cadinene and eucalyptol as the main compounds of the volatile extract.

Concerning the use of natural products to protect plants from parasitic fungi, Chàvez-Airas et al. [19] evaluated, on cape gooseberry, the elicitor effects of jasmonic acid, brassinosteroids, salicylic acid and a commercial resistance elicitor based on botanical extracts against *Fusarium oxysporum* disease. The results highlighted that all the treatments decreased the disease spread, reducing its severity index. Moreover, the treatments favoured stomatal conductance, water potential, biomass production and improved the photosynthetic performances of treated plants, compared to non-treated infected counterparts.

Among the techniques used for eco-friendly weed management, site-specific herbicide distribution should be mentioned. This technique aims to distinguish, through a stereo vision system, crops from weeds to allow a selective herbicide application and site-specific weed management. Dadashzadeh et al. [20] applied this technique in a densely cultivated rice field using artificial neural networks and two metaheuristic algorithms [particle swarm optimization and the bee algorithm] to optimize the neural network’s performance in selecting the most compelling features and classification. Their results proved that their algorithms were extremely sensitive and this technique, once developed, could powerfully help in minimizing the use of synthetic herbicides on crops.

The final original article was focused on the chemical profiling of volatiles released from pine stands infested by the pine processionary moth [21]. The work aimed to understand the variations induced by the infestations and identify those metabolites with a protective activity. The results could be beneficial for producing natural formulations with repellent activities against this widespread pine pest.

The last manuscript published on the special issue was a literature survey on *Thricoderma* sp. [22], which received in less than one year more than thirty citations in peer-reviewed manuscripts. The authors in this review focused on the plant-*Trichoderma*-pathogen triangle, highlighting the cost-effective and eco-friendly biocontrol activity of this fungus. In particular, the review summarizes the biological control activity exerted by *Trichoderma* spp., highlighting the recent progress in determining the ecological significance of *Trichoderma* at both molecular and biochemical levels. Finally, the authors highlighted the benefits of the symbiosis to the plant host concerning biochemical and physiological mechanisms.

The Special Issue’s editors are grateful to all the authors who contributed to the issue with their priceless contribution, as well as MDPI’s collaborators for their valuable support.
